# Influence of Contact Plateaus Characteristics Formed on the Surface of Brake Friction Materials in Braking Performance through Experimental Tests

**DOI:** 10.3390/ma14174931

**Published:** 2021-08-30

**Authors:** Rafael Lucas Machado Pinto, Juan Carlos Horta Gutiérrez, Robson Bruno Dutra Pereira, Paulo Eustáquio de Faria, Juan Carlos Campos Rubio

**Affiliations:** 1 Mechanical Engineering Department, Federal University of Minas Gerais, Belo Horizonte 31270-901, Brazil; horta@demec.ufmg.br (J.C.H.G.); juan@demec.ufmg.br (J.C.C.R.); 2 Department of Production Engineering, Federal University of Ouro Preto, João Monlevade 35931-008, Brazil; 3 Department of Mechanical and Production Engineering, Federal University of São João del-Rei, São João del-Rey 36301-158, Brazil; robsondutra@ufsj.edu.br; 4 Department of Production Engineering, Federal University of Minas Gerais, Belo Horizonte 31270-901, Brazil; paulofaria@ufmg.br

**Keywords:** friction material, braking, wear mechanisms, tribological contact, fade effect

## Abstract

This work applies a procedure for analysis and characterization of the surface of brake friction materials, correlating them with the tribological and thermal properties achieved in different vehicle braking conditions. Experiments were performed in a vehicle under two real conditions of braking operation, simulated flat track descent and emergency braking. Characteristics of the plates formed on the surfaces of the friction materials were analyzed by scanning electron microscopy (SEM) and correlated with the performance during braking, as measured by the coefficient of friction at the interface of the friction pair and temperature. As a result, the formation of the primary and secondary plateaus in these two different braking operating conditions was observed, and the relationship between the characteristics of the plateaus formed on the surface and the surface roughness parameters and performance measurements during braking.

## 1. Introduction

According to Machado Pinto et al. [1], automobiles and transportation vehicles have undergone several changes and improvements over the years, aiming to provide more comfortable and safer locomotion. These technological advances experienced by the automotive sector have allowed vehicles to become increasingly faster and with higher load-carrying capacities, which has also led to the need for the design and manufacture of more efficient brakes.

As described by Singh et al. [2], Nogueira [3], and Liu [4], the demands on friction behavior in brake systems are high and manifold. The coefficient of friction must be relatively high and, most importantly, remain stable regardless of temperature, humidity, time of use, degree of wear and corrosion, presence of dirt and road water spray, etc. According to Bosh [5] and Limpert [6], brake systems are intended to reduce or maintain the speed of a vehicle, bring it to a standstill, or keep it stationary. As mentioned by Barros et al. [7], Kakad and Moree [8], and Ma et. al. [9], the operation of automotive friction brake systems occurs as a function of contact between a fixed element (brake pad or lining) and a rotating element (brake disc or drum). The latter component is connected to the vehicle’s wheel. Frictional contact between the pad and the disc produces the force necessary to reduce the vehicle’s speed, converting kinetic energy into heat and also vibration, and noise. As the surfaces are subject to sliding movements, they suffer wear. Thus, periodic replacement of brake pads is necessary.

Excessive heating during the braking process can result in brake system malfunction and, consequently, reduced vehicle safety. One of these thermal problems related to brake systems is the braking fade effect, characterized by the momentary loss of friction between disc and pad due to the temperature rise of the systems during the braking process. According to Rehkopf and Halderman [10], Limpert [6], Osterle et al. [11], Mutlu [12], and Federici et al. [13], among others, the fade effect can be attributed to chemical transformations that generally occur under higher temperature conditions (especially above 300 °C). Significant chemical changes occur in the friction material at elevated temperatures, such as the degradation of phenolic resin by oxidation and burning of volatile materials. Some oxides have lubricating properties and can cause a reduction in friction, making the fade effect more noticeable.

According to Xiao et al. [14] and Zhang et al. [15], when the brake is applied, the rough surfaces of the brake pad and brake disc are subjected to sliding. As the brake disc, which has much higher hardness than the pad, rotates at high speed, some friction materials are removed by cutting, forming wear debris. As described by Eriksson, Bergman, and Jacobson [16] and Leonardi et. al. [17], the friction layer (or third body) consists of a thin layer, which is deposited on the surface of the pad and brake disc as a result of the wear process. As mentioned by Ostermeyer and Muller [18] and Seo et al. [19], the existence of the plateaus is linked to the process of generating friction pair wear debris during braking, which strongly influences the friction and wear behavior of brake pairs. Osterle and Urban [20] point out that compact friction layers comprise a nanocrystalline microstructure and are observed on both pad and disc surfaces. Since these areas are smooth and the surrounding areas are rough, the authors state that the former are the load-bearing contact areas that possess frictional power. Osterle et al. [11] and Osterle and Urban [20], point out that the friction layer determines the friction characteristics during a braking process.

In this study, braking tests were conducted on a real vehicle under two different operating conditions: simulated downhill driving on a flat track and emergency braking. These experiments were conducted to analyze the properties of the brake friction material, specifically regarding the characterization of the contact plateaus formed on the surface of brake friction materials and the 2D roughness parameters, correlating them with the tribological and thermal properties achieved under different vehicle braking conditions. To analyze the plateau characteristics, scanning electron microscopy (SEM) and energy dispersive spectroscopy (EDS) were used. To analyze the relationships between the collected variables, the statistical techniques t-test and main effect plots were utilized. Wear mechanisms were identified on the sample surfaces and related to the results obtained.

## 2. Materials and Methods

### 2.1. Vehicle Tests Description

Friction vehicle tests were performed in a test track of an automaker present in the Brazilian marketplace. The vehicle used in the tests is equipped with the brake disc in the front axle and with the drum brake in the rear axle. The instruments and sensors utilized for temperature and friction coefficient data acquisition are described next.

### 2.2. Instruments Utilized in Vehicle Tests

To control the hydraulic pressure of the brake, it was installed an HBM full-bridge extensometer sensor (Manufacturer HBM, Darmstadt, Germany) model P8AP with a measuring range from 0 to 3000 psi. This sensor was installed in the hydraulic tubing of the brake system. To control the temperature of the discs and pads, it employed thermocouples type K (Cr-Al) with 3 mm of diameter and dry tip and fiberglass insulated, to avoid fracture and favors assembly, as it is performed directly in the vehicle. These thermocouples were attached to the center of the brake pad through a hole. To control the deceleration of the vehicle, it was utilized a uniaxial accelerometer from Silicon Designs Inc.—Advanced Accelerometers (Kirkland, DC, USA), model SDI 2220-002, with a measurement range from 0 to 2 g.

**Figure 1:**
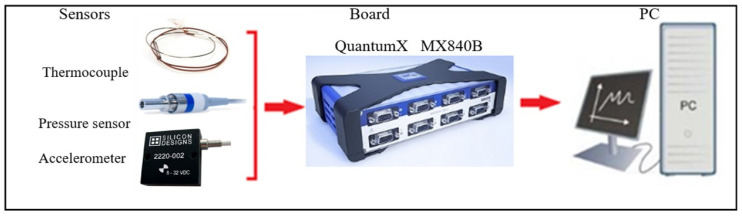
Diagram of the data acquisition system for vehicle experiments.

The data acquisition system applied in the experimental tests is composed of the thermocouples, the pressure sensor, the accelerometer, and the acquisition board. The diagram of the data acquisition system is illustrated in Figure 1.

The data acquisition of the signals measured by the sensors was performed using a Quantum X module, model MX840B from HBM (Darmstadt, Germany), with FastEthernet (RJ-45) connection. The post-processing and signal analysis was performed using the Catman Easy software, also from HBM (Darmstadt, Germany). 

### 2.3. Proceeding for the Tests Performed

The vehicle tests were performed in two distinct operational conditions:

(a) Test 1—Simulated downhill (simulated in flat track): It was performed 3 brake cycles, each one consisting of 60 brakings, with speed during braking nearby 40 Km/h. Each cycle duration was of approximately 20 min, and the average time interval between two consecutive brakings was 20 s. The brake system cools down naturally after completing a braking cycle. The next cycle starts when the system is cooled down to 100 °C.
(b) Test 2—severe braking condition on a flat surface—emergency braking: It was performed three braking cycles with 10 successive breakings, with a speed reduction of 80 Km/h, with maximum deceleration and maximum speed recovery. In these tests, the heating rate of the discs and friction materials is high, and there is not enough time for the natural cooling of the system. The brake system cools down naturally after completing a braking cycle. The next cycle starts when the system is cooled down to 100 °C.

These configurations of experimental tests in vehicles were defined because they meet the requirements of Contran Resolution 519/2015 (ABNT NBR 10966 standard), which deals with the approval of friction materials for automotive brakes in Brazil. These tests were conducted on the test track of a large vehicle manufacturer, whose name will not be disclosed in this study for reasons of secrecy and industrial secrets.

### 2.4. Experimental Design of the Vehicle Tests

In this study, the two-sample *t*-test was performed to test the difference between a simulated downhill and emergency braking. The response variables were split into three groups: breaking performance metrics, plateaus properties, and roughness parameters of the sample surfaces. The statistical software Minitab 17 (State College, PA, USA) was implemented to design and conduct the analysis. More details about this software can be found in [21]. Table 1 presents the variables of each group.

The responses considered to measure the performance of the vehicle braking system were the friction coefficient at the interface between disc and pads, and the temperature. The responses related to the plateau were obtained from microscopic analysis of the brake pad surface. A scanning electron microscope (SEM) from TESCAN (Libušina, Czech Republic), model VEGA 3, was used. The plateau measured parameters were the area of the plateaus, the average number, and the average length of the plateaus. These parameters were measured using the software of microstructural analysis Quantikov 16.01, considering SEM images with 25× magnification. Three images for each test type were analyzed. The Quantikov 16.01 (São Paulo, Brazil) software includes modules for digital image processing, geometric quantification, graphics generation, and hypertext. More details about this software can be seen in Pinto [22].

**Figure 2:**
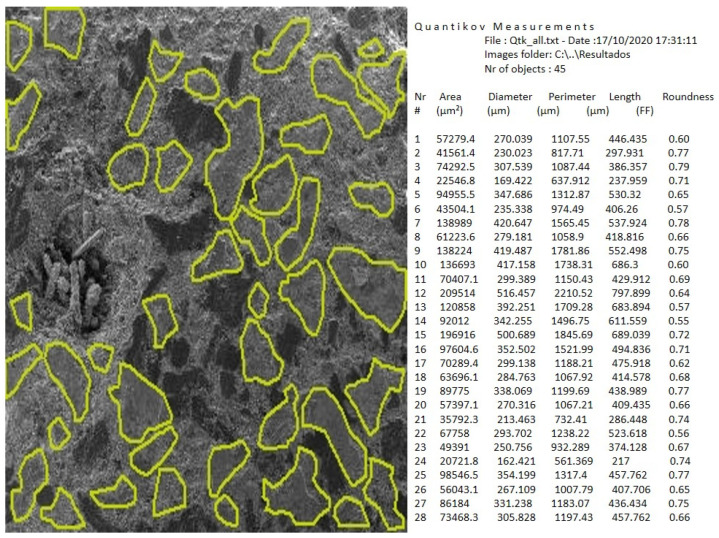
Identification of the contact plateaus using the Quantikov software and the output with the generated geometric parameters.

Figure 2 illustrates one of the images that were analyzed using the Quantikov software and an example of output provided by this software. In the group of variables related to surface roughness aspects, the selected parameters were: Ra, Rz, and Rt. Ra is the arithmetic mean of the deviations of the surface profile, defined over a sample length I. Rz is the average of 5 amplitudes taken over the evaluation lengths. Rt is the maximum roughness height, that is, the maximum amplitude between the highest peak and the deepest valley at the evaluation length. Three equally spaced measurements of the roughness parameters were taken on each sample. A Taylor Hobson Precision (Manufacturer Taylor Hobson, Leicester, Great Britain) model Surtronic 25 roughness meter was utilized.

### 2.5. Description of the Friction Materials Tested

**Figure 3:**
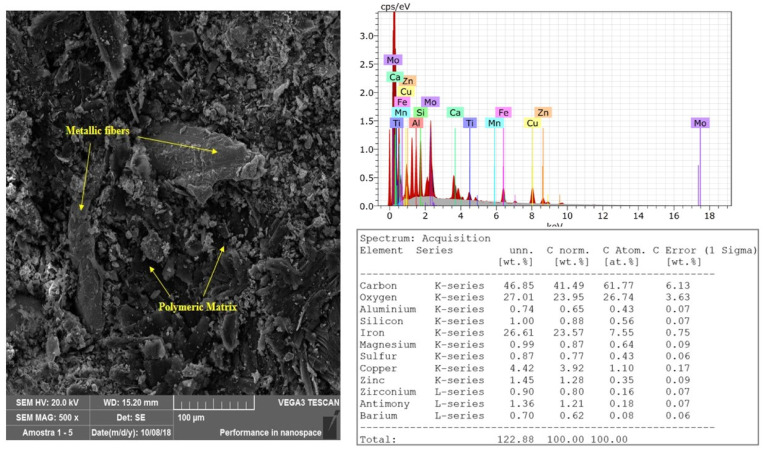
SEM images—500×—and EDS estimation of the chemical composition.

Figure 3 illustrates the SEM image of a brake pad, with the magnification of 500×. As the material was not yet subjected to the friction process, there is no plateau presence with which it would be possible to see irregularities. There are also no abrasion marks. It is possible to see with more details of the pad constituents, especially the metal fibers. The dark regions are the polymeric matrix of the material. The semi-quantitative EDS analysis of the new friction pad is also illustrated in Figure 3. The main elements composing the pad are carbon, oxygen, iron, and copper. This pad is made of a typica non-asbestos organic (NAO) friction material, which according to Birch [23], is used in light vehicles and in markets where the comfort in the braking operation is prioritized. All brake discs tested were made of gray cast iron.

## 3. Results and Discussion

**Figure 4:**
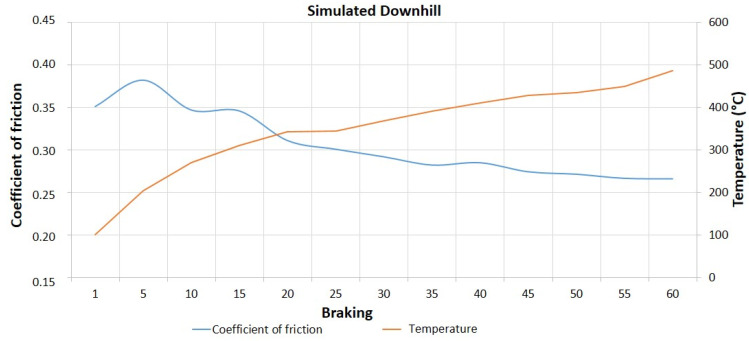
Coefficient of friction and temperature vs. number of brakes—ΔV = 40 Km/h.

**Figure 5:**
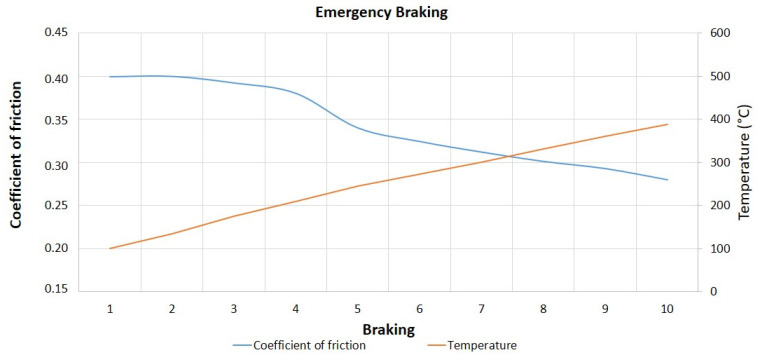
Coefficient of friction and temperature vs. number of brakes—ΔV = 80 Km/h.

Figure 4 and Figure 5 illustrate, respectively, the graphs of coefficient of friction and temperature, as a function of the number of braking, for the simulated downhill tests with a speed reduction of 40 Km/h and the first cycle of emergency braking, with a speed reduction of 80 Km/h. For the simulated downhill tests, it is possible to note that the reduction of the coefficient of friction begins to occur approximately from braking 5. For the emergency braking tests, the decrease of the coefficient of friction is accentuated from braking 4. In both tests, the temperature increases progressively as a function of the number of braking actions, and the decrease in the coefficient of friction values, characterizing the fade effect, occurs for temperature values around 250 to 300 °C.

The fade effect, as described by Rehkopf and Halderman [10], Limpert [6], Osterle et al. [11], Mutlu [12], and Neis [24], among others, can be attributed to chemical transformations that generally occur under higher temperature conditions (degradation of phenolic resin and burning of volatile materials). Deng et al. [25] point out that when the surface temperature reaches values high enough for the surface material to be thermally decomposed, there will be a rapid decrease in the coefficient of friction and, as a consequence, an increase in the wear rate.

Table 2 shows the descriptive statistics of the experiments for the response variable coefficient of friction. It can be seen that the emergency braking tests presented a higher mean and minimum coefficient of friction, lower amplitude, and reached lower maximum temperature values concerning the simulated downhill tests. The standard deviation for the two types of tests presented very close values (around 0.040).

To evaluate the significance of the effects of each of the variables analyzed, the *t*-test was used. Table 3 illustrates the summary of the results obtained.

In this table, the statistical *p*-value referring to the t-test is presented for each response variable. The objective was to verify whether the two operational conditions submitted to the vehicle tests promoted significant changes in these performance variables. For all variables, the assumptions of normality and independence were met. The variance between data sets was equal for all variables, and the test was applied for independent variables and equal variances. To conclude whether the analyzed factor promotes significant changes in each response variable, one can compare the *p*-values with the significance level considered, which in this study was α = 0.05. As all *p*-values obtained were lower than the significance level, the factor analyzed promotes significant changes in all response variables. Thus, for the tests performed, the simulated saw downhill braking showed statistically significant differences in all the response variables analyzed, for a 95% confidence interval, compared to the emergency braking tests.

**Figure 6:**
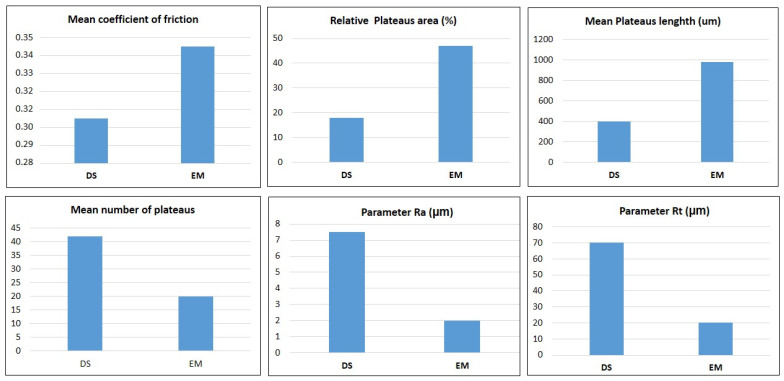
Main effect plots.

These changes can be better visualized through the graphs of main effects, illustrated by Figure 6. It is possible to notice that the emergency braking tests presented a higher average coefficient of friction, a larger average relative area with the presence of plateaus, a longer average length of plateaus, and a smaller average number of plateaus in the analyzed samples. Concerning the surface roughness parameters, it is noted that the brake pad samples submitted to the emergency braking tests presented lower values of the surface roughness parameters Ra and Rt.

**Figure 7:**
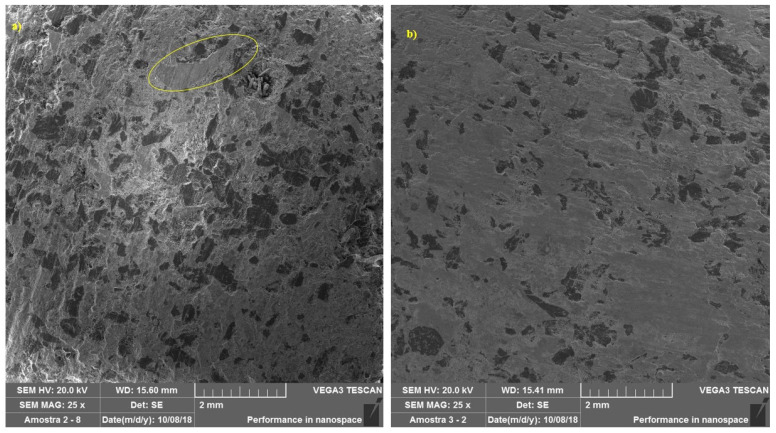
SEM images—25×: (**a**) simulated downhill; (**b**) emergency braking. Note larger plateaus and a larger relative area of the sample containing plateaus in (**b**).

The explanation for this result may be related to the measurements of the plateau characteristics: the average relative area with the presence of plateaus of the samples for these tests was larger compared to the samples of the pads used in downhill tests, as can be seen in Figure 7a,b. Since the frictional power happens through the plateaus, as described by Ostermeyer and Muller [18], this increase in contact area promotes an increase in the coefficient of friction. According to Deng et al. [25], when more asperities are deformed, worn, and fractured to form friction films, which add to the actual contact area, originating the contact plateaus, an increase in the coefficient of friction occurs. Verma et al. [26] report that primary plateaus derive from a lower removal rate of the mechanically stable and wear-resistant components of the pad material: stiff particles and tough fibers leaving the surface of the inserts when they start to wear. These primary plateaus act as barriers to the movement of the finer wear particles, which therefore tend to stop and collect at the interface with these barriers. If these particles start to stick together, this promotes the formation of secondary plateaus, as reported by Osterle et al. [27], Eriksson, Bergman, and Jacobson [16]. As mentioned by Sugozo, Mutlu, and Sugozo [28], the secondary plateaus of the third body are formed on the surface of the brake pads through the accumulation of wear residues of the pad material constituents that are compacted under the effect of normal pressure, tangential stress and heat from friction. As described by Barros et al. [29], tribofilm (friction layer or friction film) forms on the surface of the brake disc. The friction film originates from wear particles generated on the contact surface of the pad with the brake disc. It consists mainly of iron oxide.

These results observed in Figure 6 are following those described in the literature by Eriksson [30] and Eriksson and Jacobson [31]. According to these authors, considering the situation of the surface of a new brake pad, initially rougher, when the brake is applied, the primary plateaus are formed, thus increasing the possible real contact area between the pad and the disc. In addition, as the actual contact area increases and the surfaces wear, the elastic contact element increases and, consequently, the actual contact area also increases. As mentioned by the same authors, a larger actual contact area for a given pad load is expected to result in a higher coefficient of friction, as observed in the emergency braking tests. These results are also in agreement with the theory described by Straffelini [32] who states that smoother surfaces tend to have higher coefficients of friction because of the increased contact area.

The temperatures reached by the emergency braking tests were lower compared to the downhill tests and, according to Jang [33], with increasing friction temperature, the matrix resin can be softened and even carbonized to lose its adhesion strength. As a consequence, the friction films will be deformed, cracked, and fragmented to form small debris that decreases friction stability, increases wear and tear, and sometimes causes strong vibrations and loud noise. Once the surface temperature exceeds the thermal decomposition temperature of the friction material, the fade phenomenon will occur and the coefficient of friction will consequently decrease.

### Scanning Electron Microscopy (SEM) Analysis—Plateaus Formation Process

**Figure 8:**
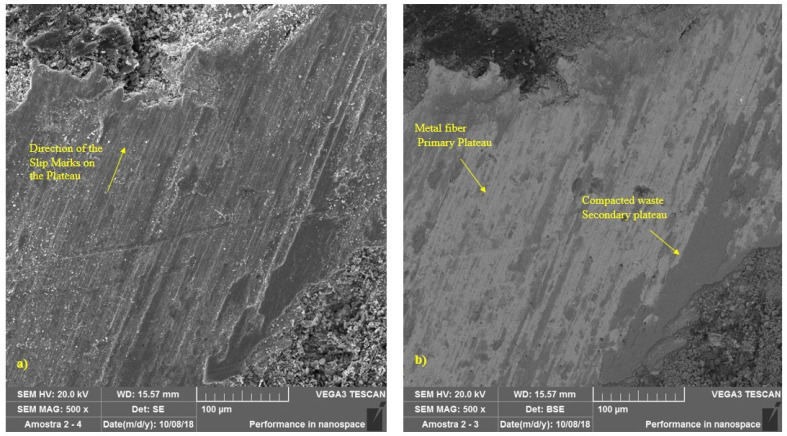
SEM images—500×—for pads under simulated downhill; (**a**) SE; (**b**) BSE.

**Figure 9:**
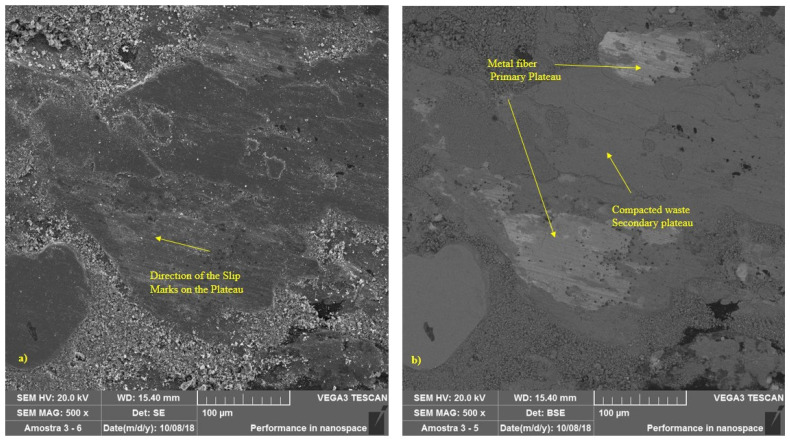
SEM images—500×—for pad under emergency braking: (**a**) SE; (**b**) BSE.

In contrast to the new material, represented by Figure 3, the formation of plateaus was observed in brake pads submitted to the two operational conditions tested. Figure 8 illustrates, at 500× magnification, the region highlighted in Figure 7a, where it is possible to better visualize the formation of primary and secondary plateaus in a sample during a simulated downhill test. Slip marks from the braking process can also be seen. It can be seen that the areas around the contact plateaus show no signs of sliding contact. Figure 9 represents a sample, also at 500× magnification, subjected to the emergency braking experiment. Note that the secondary plateau was formed from the compaction of wear debris around two metal fibers, which had previously formed primary plateaus.

**Figure 10:**
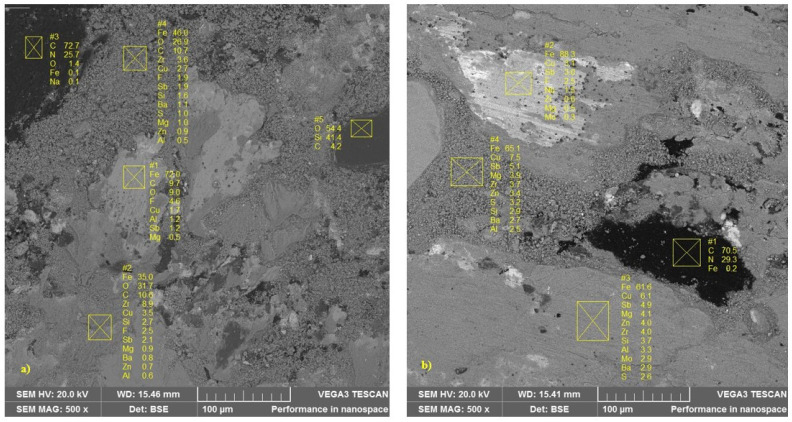
EDS analysis performed at different points: (**a**) simulated downhill; (**b**) emergency braking.

Figure 10 represents the EDS chemical characterization performed at different points on a sample submitted to the simulated downhill test (a) and emergency braking test (b). In this image, obtained by the backscattered electron method (BSE), the lighter regions represent the metallic fibers. These regions originate the primary plateaus. At these points, a higher concentration of the iron element is noted. The darker regions represent the polymeric matrix of the material, whose composition is predominantly carbon. Comparing the two samples, a higher percentage of the iron element is observed both in the primary plateaus and in the secondary plateaus in Figure 10b, which may have contributed to explaining the higher values of friction coefficient in the emergency braking tests.

**Figure 11:**
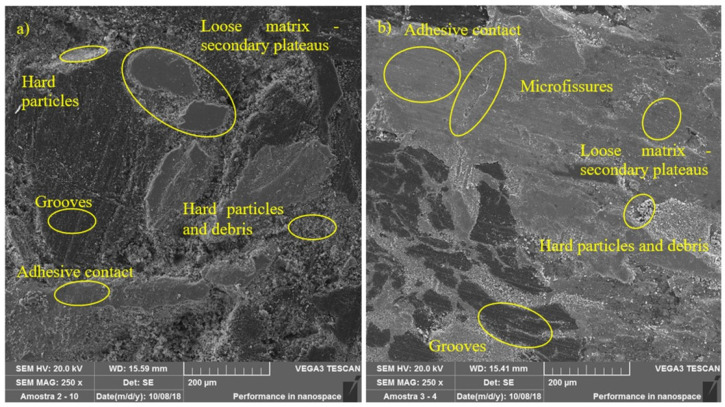
Wear mechanisms—(**a**) simulated downhill; (**b**) emergency braking.

According to Ma et al. [9] and Silvestre [34] the tribological properties of friction materials are closely related to the morphology of the worn surfaces, including wear debris, hard particles, plateaus, and microcracks. The presence of hard particles and wear debris was observed on the worn surface. The randomly (unevenly) distributed metal fibers in the polymer matrix, which constitute the pad, lead to the formation of secondary plateaus that produce a series of hard asperities and debris in the form of friction and heat. These hard asperities and debris, which are the main factor for the abrasive value, cut the worn surface, and then a series of parallel shallow channels were formed on the worn surface of the friction material. This characterizes an abrasive wear mechanism. This wear mechanism is represented in Figure 11.

As described by Wang and Liu [35] adhesion points are produced on the friction surface at high temperatures due to the metallic adhesion forces between the steel fibers and the cast iron rotating disk. Consequently, a larger adhesive contact and also wear debris appeared on the surface under the action of shear force. This contact can be increased and deepened when the hard asperities and wear debris above destroy the surface of the loose matrix (secondary plateaus) and probably constitute the main cause of adhesive wear.

On the other hand, there is a general unstable pressure and temperature field on the friction surface at high temperature, as described by Fu et. al. [36]. The different thermal expansion rates in the regions on the friction surface, due to the heterogeneous composition of the pad, could result in the formation of microcracks on the worn surface, as can be seen in Figure 11, showing typical fatigue wear characteristics.

Straffelini [32] points out that, in general, the coefficient of friction for brake applications can be expressed by the contributions from abrasive and adhesive wear forces: *μ* = *μ_abr_* + *μ_adh_*. The abrasive contribution is related to the interactions between the pad and disc asperities. Rabinowicz [37] proposes the following model for the abrasive contribution to the coefficient of friction: *μ_abr_* = *tg θ*, where *θ* is the mean angle of attack that the asperities form with the sliding surface. An estimate for *θ* is not available in this study, but according to Straffelini [32], even on very rough surfaces, its value does not exceed 10°. The remaining part of *μ* is obtained by the adhesive interaction forces between the tribological pair. This contribution, according to Rabinowicz [37], can be represented by *μ_adh_* = τ_m*_A_r_/*F_N_*, where *τ_m_* is the mean shear stress to separate the contact asperities, which is, approximately proportional to the adhesion forces, as reported by Straffelini [32], *A_r_* is the real contact area and *F_N_* is the applied load. Thus, the contribution of *μ_adh_* is expected to be minimal when the pad has its original roughness (new material) since *A_r_* is quite low. The actual contact area between the two contact surfaces is mainly determined by the contact plateaus.

As the roughness is reduced, the wear rate decreases, and the coefficient of friction increases. This behavior is determined by the progressive reduction of the abrasive component’s contribution to wear. At the same time, by reducing the roughness, the interaction of the adhesive component between pad and disk increases strongly and this induces an increase in the coefficient of friction. Comparing Figure 11, together with the analyses performed in Figure 7, it can be seen that the surface of the samples representing the emergency braking tests had a flatter and smoother appearance, where the presence of larger plateaus and a greater relative area of the sample containing plateaus can be seen. These results coincide with those found in the t-test, represented by Table 3, and by the main effects plots, illustrated by Figure 5. It was also observed lower surface roughness parameters for the samples submitted to emergency braking tests. Thus, associating the results obtained with the theories proposed by Straffelini [32] and Rabinowicz [37], it is expected that the contribution of the abrasive component of friction is more reduced in the emergency braking tests. However, because it presents more dominant adhesive surface characteristics concerning downhill braking tests, its level of relative importance becomes able to overcome its lag about the abrasive component, which makes the average coefficient of friction higher in emergency braking tests.

## 4. Conclusions

Through the experimental tests performed on a vehicle under two different conditions (downhill and emergency braking), the presence of the fade effect was observed at temperatures around 250 to 300 °C and the differences in performance during braking for these two operating conditions. Emergency braking resulted in lower temperature values at the end of braking and higher values of the mean coefficient of friction at the friction interface. It was observed that these two different braking operating conditions resulted in significant differences concerning the three groups of response variables considered. Using scanning electron microscopy (SEM), it was possible to observe that on the initially rougher surfaces of the new samples, as verified by the roughness parameters, there are no contact plateaus. For the tests in simulated downhill and emergency braking, the presence of primary and secondary plateaus was verified.

Through EDS analysis it was possible to estimate the chemical characterization of some specific points on the sample surfaces. Higher mean coefficient of friction values obtained in the emergency braking tests may be related to the higher percentage of the surface area of the samples containing plateaus, the formation of larger plateaus, and a higher percentage of iron found in the primary and secondary plateaus. It was possible to identify the main wear mechanisms resulting from the braking process, namely: abrasive wear, arising from the hard asperities and debris, generated in the tribological pair wear, which cut and formed grooves on the surface of the pads; adhesive wear, produced on the high-temperature friction surface due to metallic adhesion between the steel fibers and the cast iron rotating disk; and fatigue wear, produced due to pressure and temperature instability on the high-temperature friction surface. Emergency braking tests showed a higher mean coefficient of friction compared to downhill tests because they showed more dominant adhesive characteristics of the friction component, overcoming the lower contribution of the abrasive component in the emergency braking tests.

The experimental methodology used in this study considered the specific characteristics of the tested tribological pair as well as the boundary conditions defined in the experimental design (speed and operating conditions during braking). As a result, it was possible to analyze the relationship between the surface parameters of the friction materials and brake performance measurements. The brake pads used in this study were of the non-asbestos organic (NAO) type and gray cast-iron brake disc. This same methodology can be applied to materials with other chemical compositions, such as semi-metallic (SM) pads, as well as other operating conditions during braking.

As opportunities for the development of future work, it is suggested to perform SEM and EDS analysis also on the surface of the brake disc, in an approach similar to that conducted on the brake pads in this study, to obtain a complete analysis of the tribological pair used in brake systems.

## Figures and Tables

**Table 1:** Variables manipulated for the analysis of the vehicle experiments.

Group of Variables	Related Variables	Unit
Braking performance measurement	Coefficient of friction	-
	Temperature	°C
Measuring plateaus characteristics	Relative plateaus area	%
	Mean number of plateaus	Number of plateaus
	Mean plateau length	μm
Surface roughness parameters of the samples	Ra	μm
	Rz	μm
	Rt	μm

**Table 2:** Descriptive statistics of the experiments—response variable coefficient of friction.

Test	Mean	Standard Deviation	Minimum	Maximum	Range	Maximum Achieved Temperature
**Simulated Downhill**	0.3042	0.0405	0.2430	0.4420	0.1990	620 °C
**Emergency Braking**	0.3412	0.0401	0.2780	0.4032	0.1252	388 °C

**Table 3:** Summary of the *t*-test results.

Response Variables	*p*-Value
**Braking Performance Measurement**	
Coefficient of Friction	0.004
**Measuring Plateaus Characteristics**	
Relative plateaus area	0.038
Mean number of plateaus	0.024
Mean plateau length	0.024
**Surface roughness parameters of the samples**	
Ra	0.027
Rz	0.031
Rt	0.044

## Data Availability

Data available on request due to restrictions for industrial and marketing confidentiality.
